# Omics BioAnalytics: an RShiny application for multimodal biomarker panel discovery and assessment

**DOI:** 10.1093/bioadv/vbaf307

**Published:** 2025-11-27

**Authors:** Josh Dyce, Lea Rieskamp, Scott J Tebbutt, Bruce M McManus, Amrit Singh

**Affiliations:** Department of Anesthesiology, Pharmacology and Therapeutics, The University of British Columbia, Vancouver, BC V6T 1Z3, Canada; Room 166, Centre for Heart Lung Innovation, St. Paul's Hospital, Vancouver, BC V6Z 1Y6, Canada; Providence Research, Providence Health Care, Vancouver, BC V6Z 2K5, Canada; Department of Biology, Lund University, Lund, SE-223 62, Sweden; Room 166, Centre for Heart Lung Innovation, St. Paul's Hospital, Vancouver, BC V6Z 1Y6, Canada; Providence Research, Providence Health Care, Vancouver, BC V6Z 2K5, Canada; Department of Medicine, Gordon & Leslie Diamond Health Care Centre, The University of British Columbia, Vancouver, BC V5Z 1M9, Canada; Room 166, Centre for Heart Lung Innovation, St. Paul's Hospital, Vancouver, BC V6Z 1Y6, Canada; Prevention of Organ Failure (PROOF) Centre, Vancouver, BC V6Z 2K5, Canada; Department of Pathology and Laboratory Medicine, The University of British Columbia, Vancouver, BC V6T 2B5, Canada; Department of Anesthesiology, Pharmacology and Therapeutics, The University of British Columbia, Vancouver, BC V6T 1Z3, Canada; Room 166, Centre for Heart Lung Innovation, St. Paul's Hospital, Vancouver, BC V6Z 1Y6, Canada; Providence Research, Providence Health Care, Vancouver, BC V6Z 2K5, Canada

## Abstract

**Motivation:**

Machine learning offers a powerful approach for building predictive models from high-dimensional molecular data. Omics technologies such as transcriptomics, proteomics, and metabolomics quantify thousands of molecules simultaneously, providing deep insights into disease biology. Integrating multiple modalities can enhance predictive performance, as shown in histology-omics and holter-omics applications. To support streamlined, reproducible, and user-friendly multimodal analytics, we developed Omics BioAnalytics, an R Shiny platform for unified analysis, integration, and interpretation of diverse omics datasets.

**Results:**

Omics BioAnalytics performs late integration using ensembles of elastic net models trained independently on each modality, with predictions averaged across datasets. The platform provides interactive dashboards for metadata exploration, exploratory analyses, differential expression, gene set analysis, and biomarker discovery. Results are visualized through dynamic plots and downloadable reports, ensuring transparent and reproducible workflows. A unique feature is the integrated multimodal Alexa Skill, which enables voice-based querying and rapid visualization. Together, these web and voice-enabled tools offer accessible and reproducible multimodal analytics for biomedical researchers, supporting the discovery of molecular signatures, predictive biomarkers, and therapeutic targets.

**Availability and implementation:**

All source code, public datasets, video walkthroughs, and the deployed application are available at: https://github.com/CompBio-Lab/omicsBioAnalytics.

## 1 Introduction

Machine learning has emerged as a powerful tool for developing prediction models for clinical outcomes using molecular (omics) data. Omics technologies such as transcriptomics, proteomics, and metabolomics enable the simultaneous quantification of thousands of molecules from biological specimens, offering a comprehensive view of disease states. We and others have shown that integration of multiple modalities improves predictive performance, for example, using histology-omics to predict survival outcomes (Chen *et al.* 2022) and holter-omics to predict heart failure hospitalizations (Singh *et al.* 2019a). A variety of strategies exist for integrating multimodal datasets (Cai *et al.* 2022). These include early integration (concatenation of data across modalities), intermediate integration (extracting shared sources of variation), and late integration (combining independently trained models). Among these, late integration is particularly advantageous for diagnostic model development, as it accommodates missing modalities, selects the strongest predictors from each omics type, and scales efficiently to large datasets.

**Figure 1.**
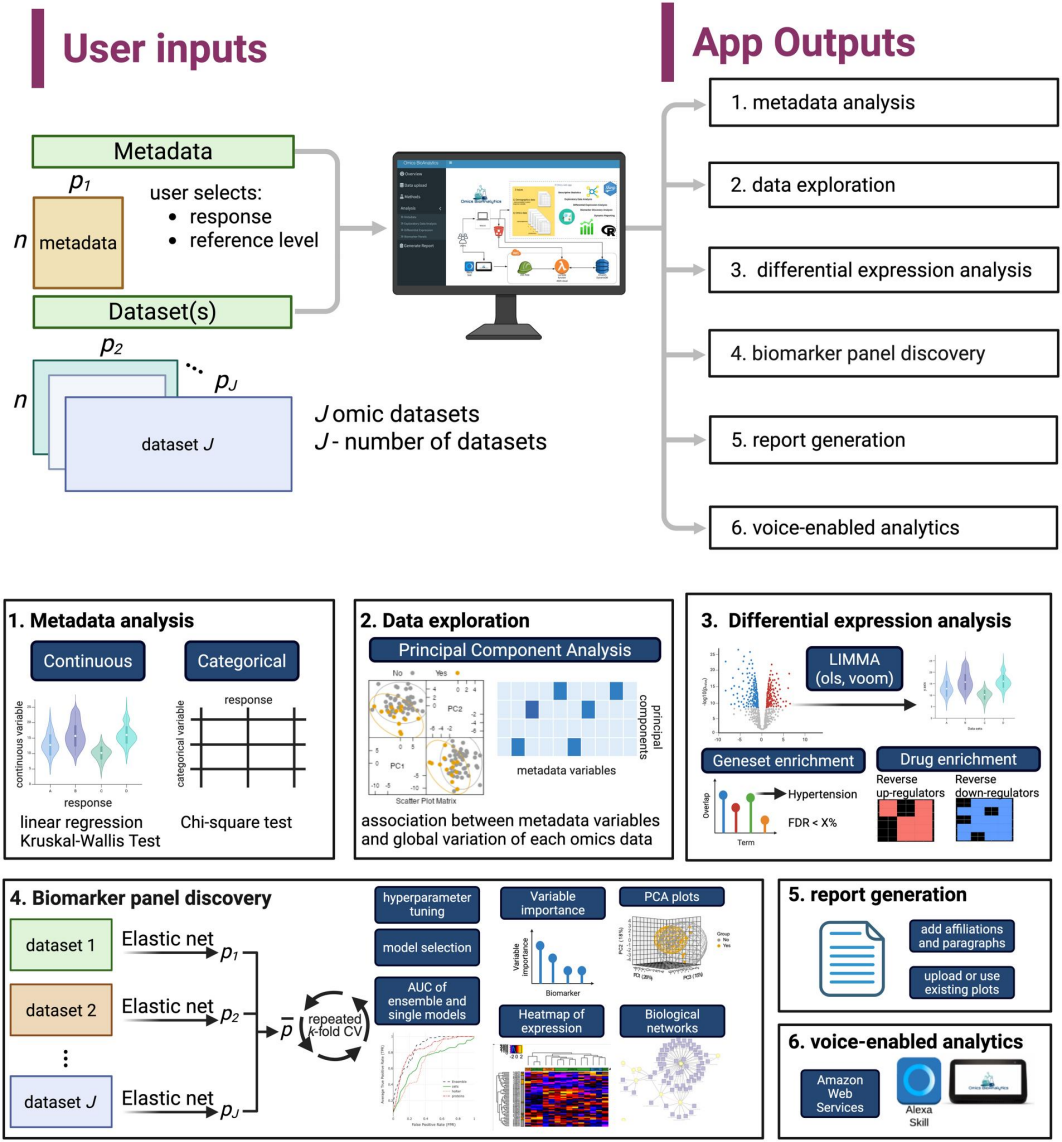
Overview of Omics BioAnalytics. The web app allows users to upload multiple datasets (omics and non-omics) data collected on the same set of samples as well as their associated metadata variables (e.g. demographic and clinical data). The user can then perform various analyses such as analysis of metadata variables (1), exploratory data analysis (2), differential expression analysis (3), and biomarker discovery analysis (4). Outputs such as figures can be downloaded and uploaded to generate a word document to summarize research findings (5). The multimodal Alexa Skill allows users to exploratory analyses on the datasets using voice-commands (6). Created with BioRender.com.

Several interactive platforms have been developed to facilitate multiomics analysis. Tools such as OmicsAnalyst (Zhou *et al.* 2021) and XOmicsShiny (Gao *et al.* 2024) provide intermediate integration, network analyses, and trend analysis. Omics BioAnalytics uniquely supports late integration, metadata analysis, report generation, and even voice-enabled analytics (see Table 1, available as supplementary data at *Bioinformatics Advances* online, for comparison with existing tools). Specifically, late integration in Omics BioAnalytics is implemented through ensembles of elastic net models trained independently on each omics dataset, with predictions averaged across modalities. This ensemble strategy has been shown in our previous work to perform competitively with intermediate integration approaches such as DIABLO (Singh *et al.* 2019b). The biomarker discovery pipeline leverages the caret R package for hyperparameter tuning, with model selection based on repeated cross-validation. Here, we present Omics BioAnalytics (Fig. 1), an extensible R Shiny platform that unifies descriptive statistics of metadata, multimodal integration, gene set analysis, ensemble learning, and dynamic report generation. Importantly, we complement the web application with a multimodal Alexa Skill, enabling voice-based interaction with complex datasets. Together, these innovations provide reproducible, accessible, and interpretable workflows for biomedical and life-science studies, from data preprocessing through to biomarker interpretation and report generation.

## 2 Methods

### 2.1 Data formats

Metadata, which includes demographic variables such as age and sex, is typically organized in spreadsheets with variables (*p*) along the columns and individual observations (*n*) along the rows, resulting in an *n × p* format. Omics BioAnalytics standardizes all inputs to the *n × p* orientation, assuming that the metadata file and all uploaded omics data files share the same sample ordering. Users first upload a metadata file, select a categorical response variable (e.g. disease status) and its reference level, and then upload one or more omics datasets. At a minimum, the application requires two files: a metadata file and an omics data file where samples are rows and variables are columns. However, users may supply multiple omics datasets corresponding to the same *n* samples. Maintaining consistent sample order across all files ensures proper data alignment during analysis.

### 2.2 Metadata exploration

In clinical research papers, the first table (Table 1) is used to summarize key demographic and clinical variables of study participants/patients, typically stratified by study group (e.g. treatment vs. placebo or disease vs. healthy). This is analogous to the user-selected response variable in Omics BioAnalytics. To facilitate metadata exploration, the application enables users to assess associations between each metadata variable and the chosen response variable. In the Metadata tab, metadata variables are automatically categorized as numerical or categorical. Continuous variables can be analysed using either linear regression or the Kruskal–Wallis test, with the gvlma R package employed to validate regression model assumptions. The application presents detailed R outputs from the lm and kruskal.test functions, including test statistics and *P* values, along with descriptive summaries such as means and standard deviations for regression, or minimum, median, and maximum values for nonparametric tests. Categorical variables are displayed as counts and percentages, with the chisq.test function used to assess associations with the response variable. Together, these analyses provide a comprehensive, interactive version of Table 1, offering users a structured overview of cohort characteristics and potential relationships within the metadata.

### 2.3 Exploratory data analysis

Exploratory data analysis involves performing Principal Component Analysis (PCA) on each *n x p_j_* omics dataset, where *p_j_* represents the number of variables in the *j*th dataset, *j=1,…,J*. The results from each omics dataset are displayed in separate interactive tabs. For each dataset, users can select up to five principal components, which are visualized using a scatter plot matrix (score plot) where samples are coloured according to the response variable specified by the user during data upload. The proportion of variance explained by each component, as obtained from the prcomp function in R, is also presented. To interpret sources of variation, the association between metadata variables and PC scores is assessed through linear regression, where PC scores are regressed onto categorical or continuous metadata variables. The resulting ANOVA *P* values are visualized as a heatmap, highlighting significant relationships between metadata and major axes of variation. Users also have the option to apply the Benjamini-Hochberg false discovery rate (FDR) correction for multiple testing. Thus, for each omics dataset, a dynamic dashboard is generated, illustrating the *k*-selected principal components through colour-coded scatter plots and a heatmap that attributes major sources of variation to known sample characteristics such as batch or response groups.

### 2.4 Differential expression and gene set analysis

For each omics dataset provided, a separate dashboard is dynamically generated to display the results of the differential expression analysis, including an interactive volcano plot [−log_10_(*P* value) vs. log_2_(fold-change)] that specifies the significant and not significant variables based on a user controlled FDR interactive slider. Clicking on the points of the volcano plot displays each individual variable using a violin plot. Since the volcano plot only displays pairwise comparisons, for response variables with multiple categories, the user can select which pairwise comparison to view as well as the type of methodology to use including ordinary least squares (OLS), limma (suited for microarrays) and limma-voom (suited for RNA-Seq data) (Ritchie *et al.* 2015). For omics datasets in which human gene symbols are detected (e.g. transcriptomics, proteomics), significant variables (based on a user-selected FDR) are used to determine biologically enriched pathways (depicted using interactive dot plots) using EnrichR for *Homo sapiens* only (Xie *et al.* 2021). The up- and down-regulated significant genes are used to also identify drug candidates that reverse these expression profiles (depicted using interactive heatmaps). Pathway analyses are based on Jensen_DISEASES, KEGG_2019_Human and WikiPathways_2019_Human gene sets, whereas the drug analyses are based on the LINCS_L1000_Chem_Pert_up and LINCS_L1000_Chem_Pert_down gene sets. The background “universe” gene list utilized for the gene set analysis is based on the variables (e.g. genes/proteins) tested in the differential expression analysis, to adjust for sampling bias (Timmons *et al.* 2015).

### 2.5 Biomarker discovery analysis

To identify biomarker panels consisting of a subset of variables from each omics dataset, binary classification models using penalized regression with the elastic net penalty (Zou and Hastie 2005) are developed for each user-specified dataset (glmnet R package). As the default distribution for glmnet is Gaussian we recommend the user to utilize normally distributed datasets for biomarker analysis. The ensemble of models is aggregated by averaging the predicted probabilities. Repeated cross-validation is used to determine the optimal values for hyperparameters based on a user-defined grid. The user-specified seed allows for reproducible data splitting during cross-validation. The performance of the optimal single and ensemble biomarker panels is depicted using receiver operating characteristic curves. The importance of the selected biomarkers is displayed using interactive dot plots. Overlaps between the single and ensemble biomarker panels are shown using an intersection plot. PCA score plots and expression heatmaps are used to depict the clustering of the samples based on the selected biomarkers in single and ensemble biomarker panels. The variables of the ensemble biomarker panel are used to perform gene set analysis (see Section 2.4 for details). The significant pathways (based on user-selected FDR threshold) are used to depict a network connecting pathways with biomarkers. Biomarkers are also connected using a Pearson correlation cut-off (also user-specified).

### 2.6 Dynamic reporting

For each analysis, figures and tables can be downloaded by the user. Similar to a manuscript, different sections can be created using markdown syntax, and figures and tables can be attached to these sections. This process results in many HTML elements that can be rearranged by drag and drop. Finally, a word document can be generated consisting of the specified headings, text, figures, and tables for dissemination.

### 2.7 Voice-based analytics

Voice-based analytics was developed using Amazon Web Services for Amazon Alexa (Fig. 1, available as supplementary data at *Bioinformatics Advances* online, for cloud architecture). The multimodal Alexa Skill is a combination of an interaction model (consisting of user utterances) and an AWS Lambda function (handler function that responds to user utterances). Upon uploading the data files on the Omics BioAnalytics web application, the user can click the Alexa button to perform voice-based analytics using the Omics BioAnalytics Alexa Skill. The web application generates a specific id, which enables the user to retrieve the analysis when using the Alexa Skill. The analysis results such as the number of samples, number of features in each omics dataset, and the list of significant variables and pathways are stored in Amazon DynamoDB [a NoSQL database from Amazon Web Services (AWS)] and figures are stored in Amazon Simple Storage Service (S3). The lambda function is used to retrieve information and figures to display on the Alexa device based on the user’s voice commands.

## 3 Results

Using public datasets we employed our application to: (i) identify compounds that reverse the gene expression patterns observed in lung cells treated with SARS-COV-2 (Blanco-Melo *et al.* 2020) (Figs. S2–S5, available as supplementary data at *Bioinformatics Advances* online) and (ii) identify multimodal biomarker panels of heart failure hospitalizations (Singh *et al.* 2019a) (Figs S6–S14, available as supplementary data at *Bioinformatics Advances* online).

## 4 Conclusion

Omics BioAnalytics is designed to reduce the barriers to bioinformatics for non-specialists by providing accessible web- and voice-based analytical tools. The use of dynamic dashboards, dynamic reporting, interactive visualizations, and voice-enabled analytics using various bioinformatics tools will enable and expedite a thorough interrogation of multimodal data.

## Supplementary Material

vbaf307_Supplementary_Data

## Data Availability

No additional data were generated as part of this research.
