# Supplementary material for: Omics BioAnalytics: an RShiny application for multimodal biomarker panel discovery and assessment
Source: Bioinform Adv. 2025 Nov 27;6(1):vbaf307. doi: 10.1093/bioadv/vbaf307 (PMC12782103; doi:10.1093/bioadv/vbaf307)
Supplement: vbaf307_Supplementary_Data [file vbaf307_supplementary_data.docx]

# Supplementary Information

**Supplementary Table S1. Compare and contrast between various features of existing web applications for multiomics data analyses.**

|  | OmicsAnalyst | XOmicsShiny | Omics BioAnalytics |
| --- | --- | --- | --- |
| Data preprocessing | **+** | **-** | **-** |
| Intermediate integration | **+** | **-** | **-** |
| Network analysis (WGCNA, PCSF) | **-** | **+** | **-** |
| Pattern and trend analysis | **-** | **+** | **-** |
| Data exploration | **+** | **+** | **+** |
| Differential expression analysis | **+** | **+** | **+** |
| Gene set analysis and/or Gene set enrichment analysis | **+** | **+** | **+** |
| Late integration | **-** | **-** | **+** |
| Drug enrichment analysis | **-** | **-** | **+** |
| Metadata analysis | **-** | **-** | **+** |
| Report generation | **-** | **-** | **+** |
| Voice-enabled analytics | **-** | **-** | **+** |


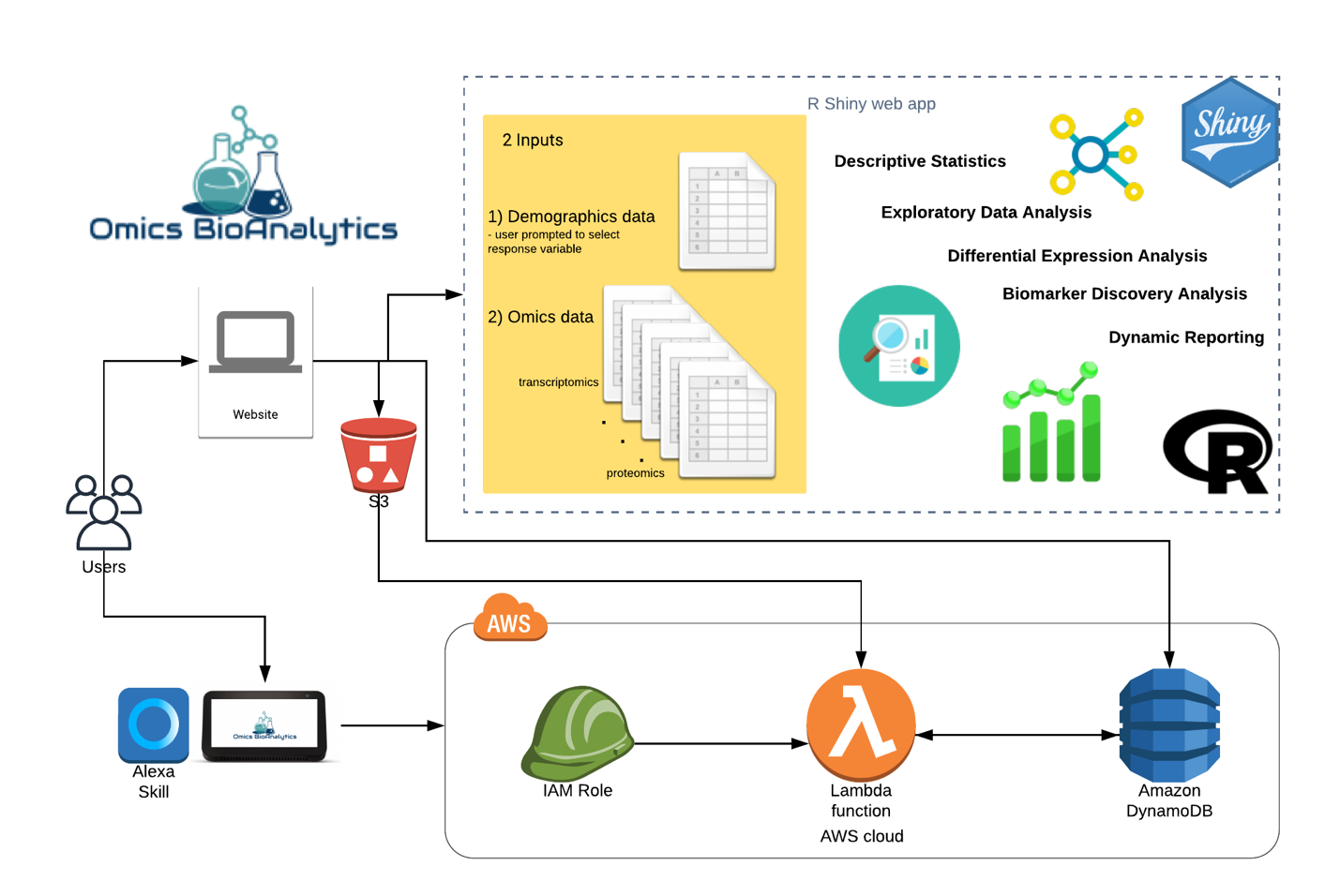


**Supplementary Figure S1. Overview of Omics BioAnalytics cloud architecture.**

All of the following analyses were performed at <https://amritsingh.shinyapps.io/omicsBioAnalytics/>. Links to download the associated datasets are also provided on the web application.

**Case Study 1: Identifying compounds that reverse the gene expression patterns observed in lung cells treated with SARS-COV-2**

*Methods:* RNA-Sequencing was performed on RNA collected from lung epithelial cells under control and infected conditions (20 samples) ([**GSE147507**](https://www.ncbi.nlm.nih.gov/geo/query/acc.cgi?acc=GSE147507)) (Blanco-Melo *et al.*, 2020). Data prefiltering was performed to remove gene transcripts with zero variance and low abundance resulting in 10,563 gene transcripts (see details for data preprocessing here: https://github.com/CompBio-Lab/omicsBioAnalytics/blob/main/inst/extdata/covid19/covid19.md).

Steps:

1. **Data upload**


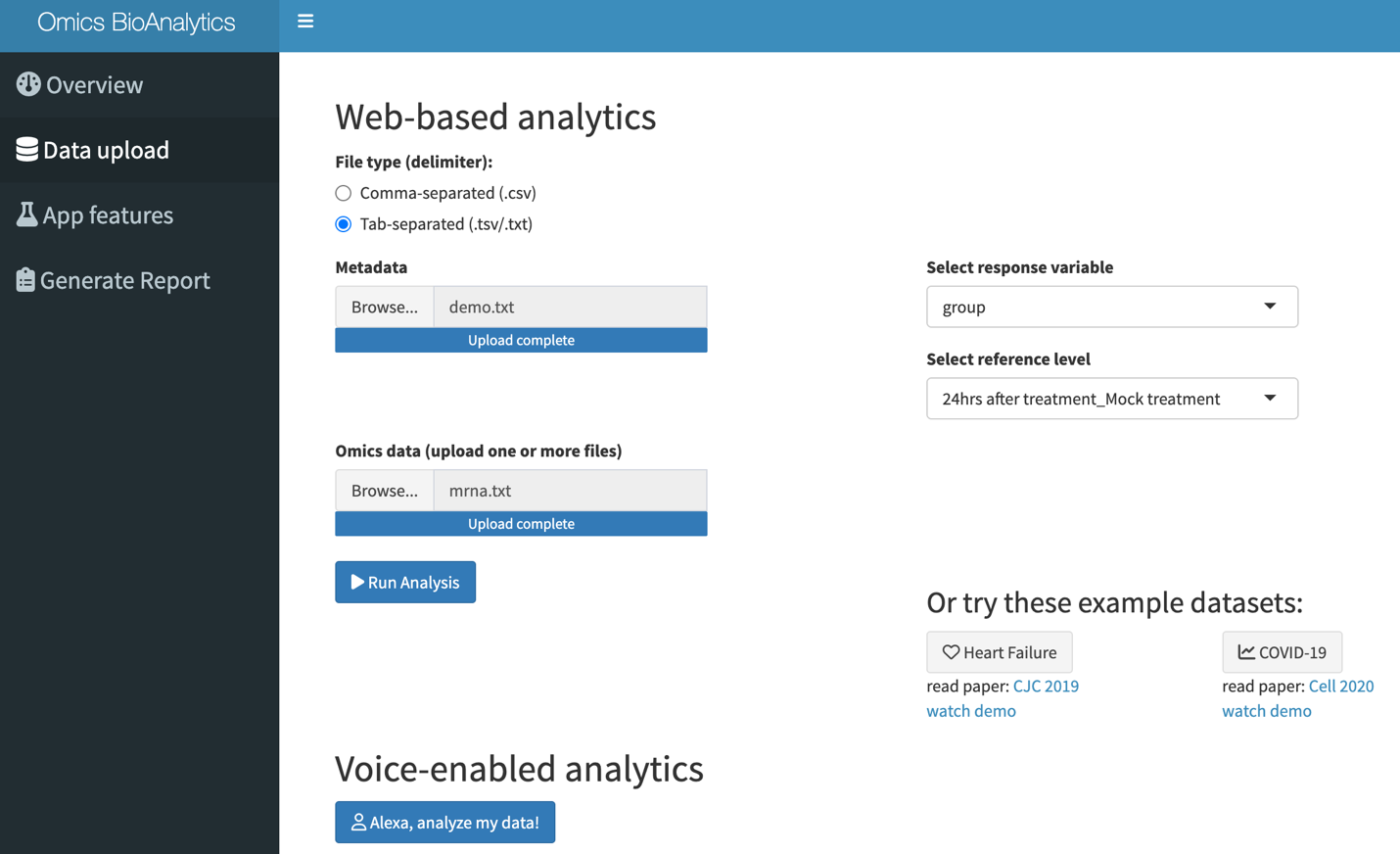


**Supplementary Figure S2. Data upload of COVID-19 datasets.** Metadata of the samples can be uploaded which prompts the user to select a response variable as well as a reference category. In this case, the response variable *group* and the reference category *24hrs after treatment_Mock treatment* were selected. One or more omics datasets can also be uploaded, in this case one text file named *mrna* was uploaded. All files have samples along the rows and variables along the columns.

1. **Differential Expression and Gene Set Analysis**


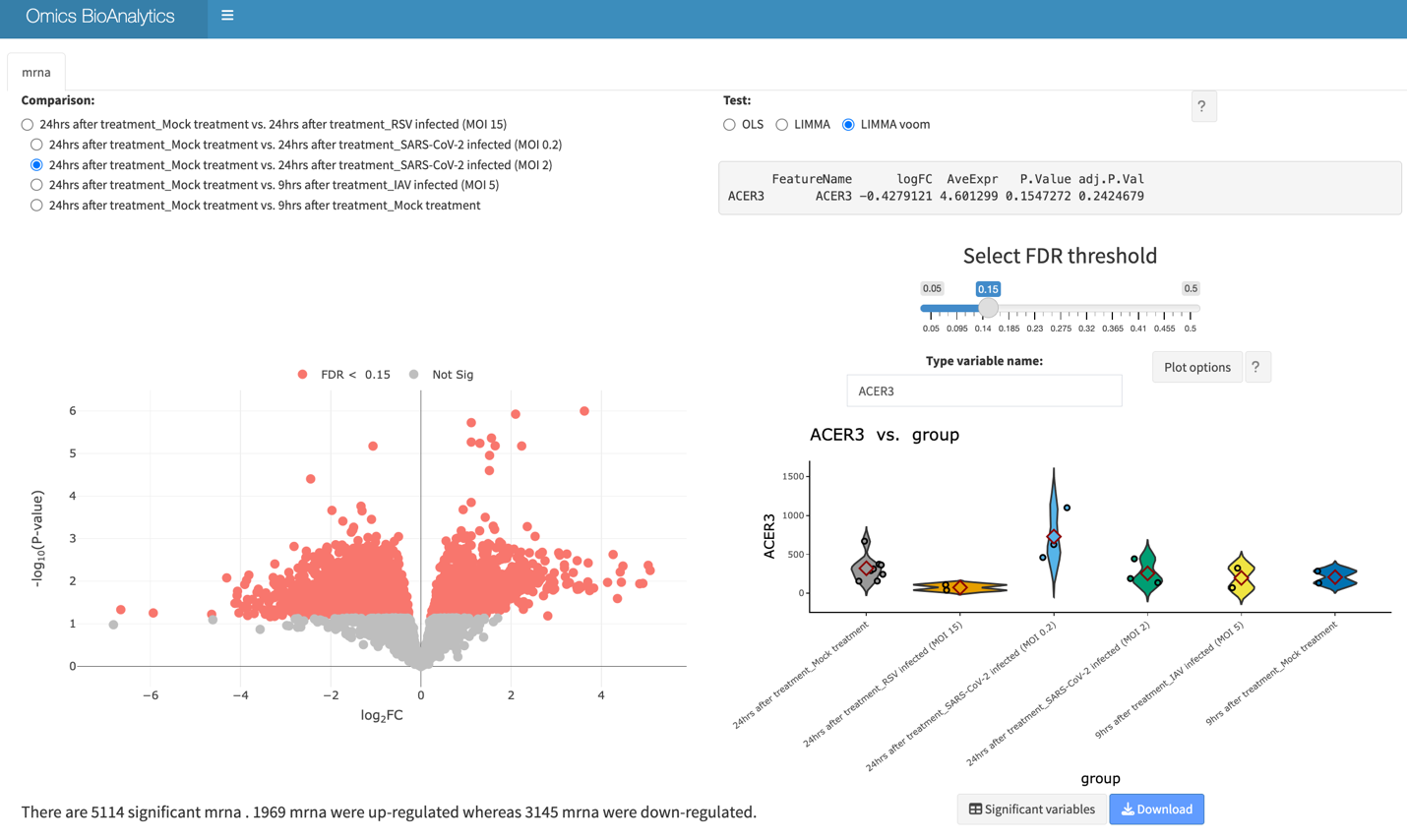


**Supplementary Figure S3: Differential expression analysis of SARS-COV-2 RNA-Seq data.** 5114 differentially expressed genes (mRNA) were identified comparing mock treatment (24h) with SARS-COV-2 infected cells at an FDR=15%. All possible pairwise comparisons can be conducted by using the radio buttons. Clicking on points on the volcano plot (left) or typing in a gene symbol (with autocompletion of variables that exist in the current dataset) can be used to plot the expression counts of a single variable (right), in this case, *ACER3* expression across treatment groups. LIMMA voom which is suited for RNA-Seq count data was used for this analysis. Users can see the list of significant variables by clicking on the *Significant variables* button or download the results for their own purposes. Plots can be customized and downloaded.


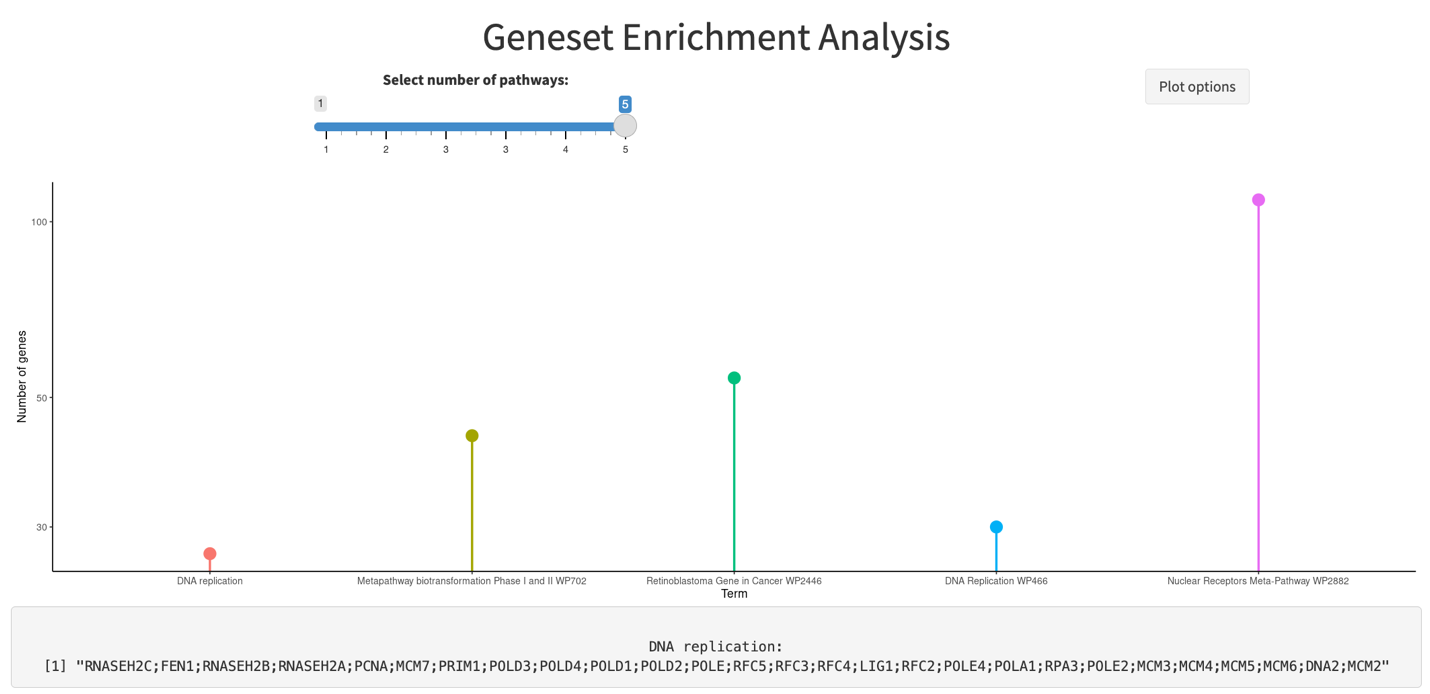


**Supplementary Figure S4: Gene set analysis using EnrichR.** For datasets in which the web application detects gene symbols, gene set analysis is automatically performed using the EnrichR using the Jensen_DISEASES, KEGG_2019_Human and WikiPathways_2019 gene set collections based on the list of significant variables from Figure S3. Five significant pathways (FDR=15%) are plotted. Clicking on the tip of each point provides additional information such as the overlap between members of the significant gene list and gene set. This data can be downloaded in a table format by clicking on the *Download Enriched Pathways* button.

A.


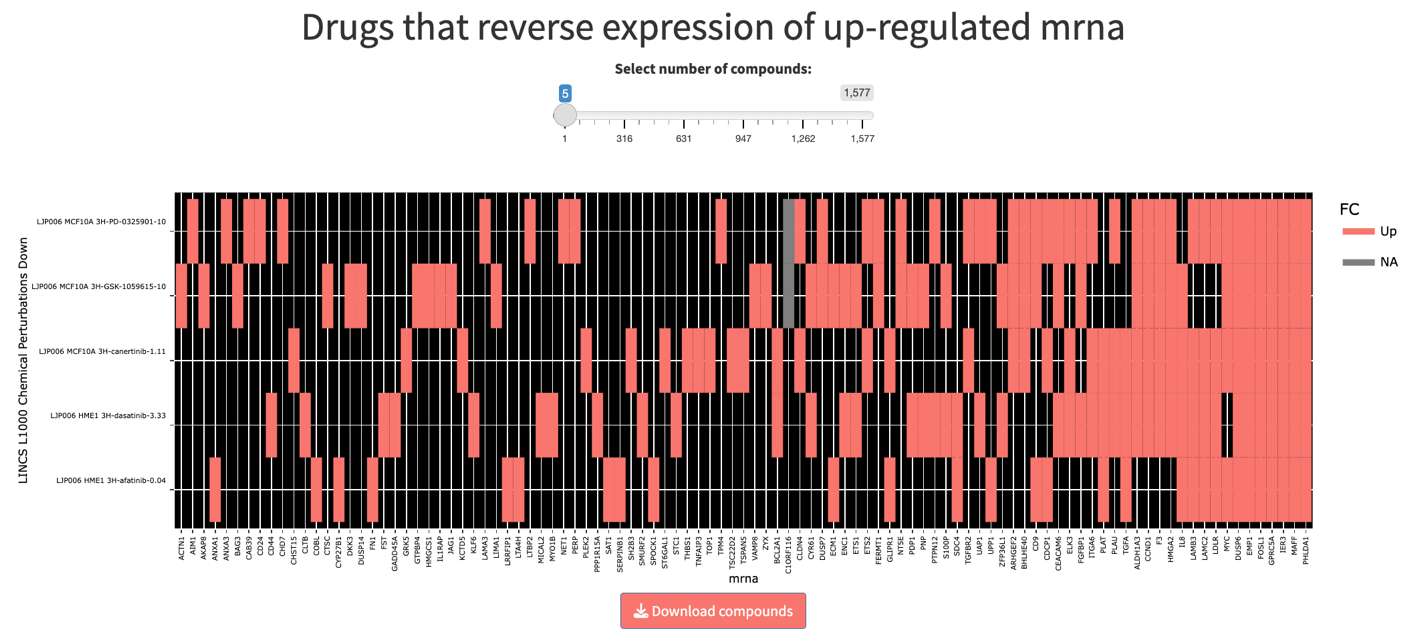


B.


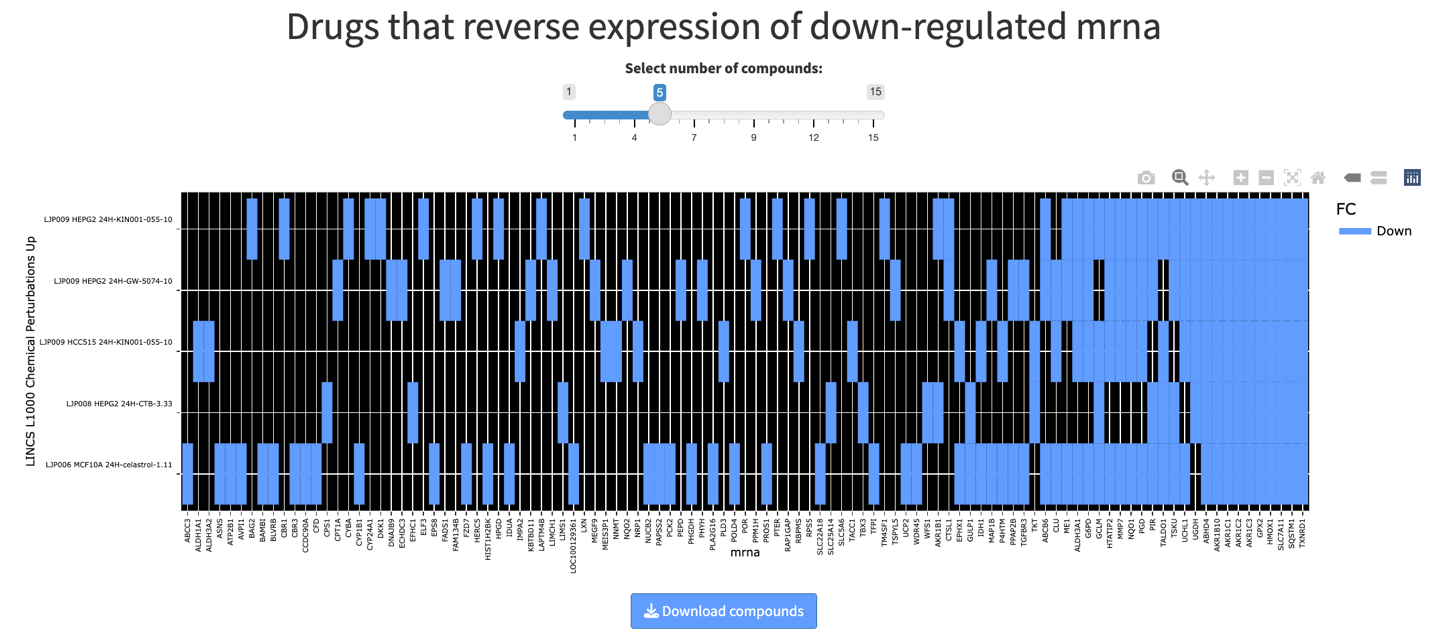


**Supplementary Figure S5: Drug enrichment analysis using EnrichR.** For datasets in which the software detected gene symbols, gene set analysis was automatically performed using EnrichR using the LINCS_L1000_Chem_Pert_down and LINCS_L1000_Chem_Pert_down gene set collections based on the list of significant variables from Figure S3.The 1969 up- and 3145 down-regulated mRNA transcripts are used to identify chemical compounds that can reverse the expression profiles (from Figure S3). **A.** Chemical compounds that decrease the expression of a significant number of up-regulated genes by SARS-COV-2. **B.** Chemical compounds that increase the expression of a significant number of down-regulated genes by SARS-COV-2. Each plot can be zoomed in further to enable better viewing of compound and gene names.

**Case Study 2: Identifying multimodal biomarker panels of heart failure hospitalizations**

*Methods:* As previously described (Singh *et al.*, 2019), blood samples were collected from 58 patients with heart failure and profiled for gene expression using microarrays (5000 gene transcripts) and protein expression using mass spectrometry (65 proteins). 29 electrical variables from Holter monitors were also collected. Cell-type frequencies were estimated using cell marker genes. Although these four omics datasets were used to identify a subset of biomarkers that were predictive of 3-month cardiac related hospitalizations, for the purposes of the web application which is on the free plan provided by shinyapps.io, we limit the biomarker analysis to three omics datasets: cells, holter and proteins. (See data compilation steps here: https://github.com/CompBio-Lab/omicsBioAnalytics/blob/main/inst/extdata/heartFailure/heartFailure.md)

Steps:

1. **Data Upload**


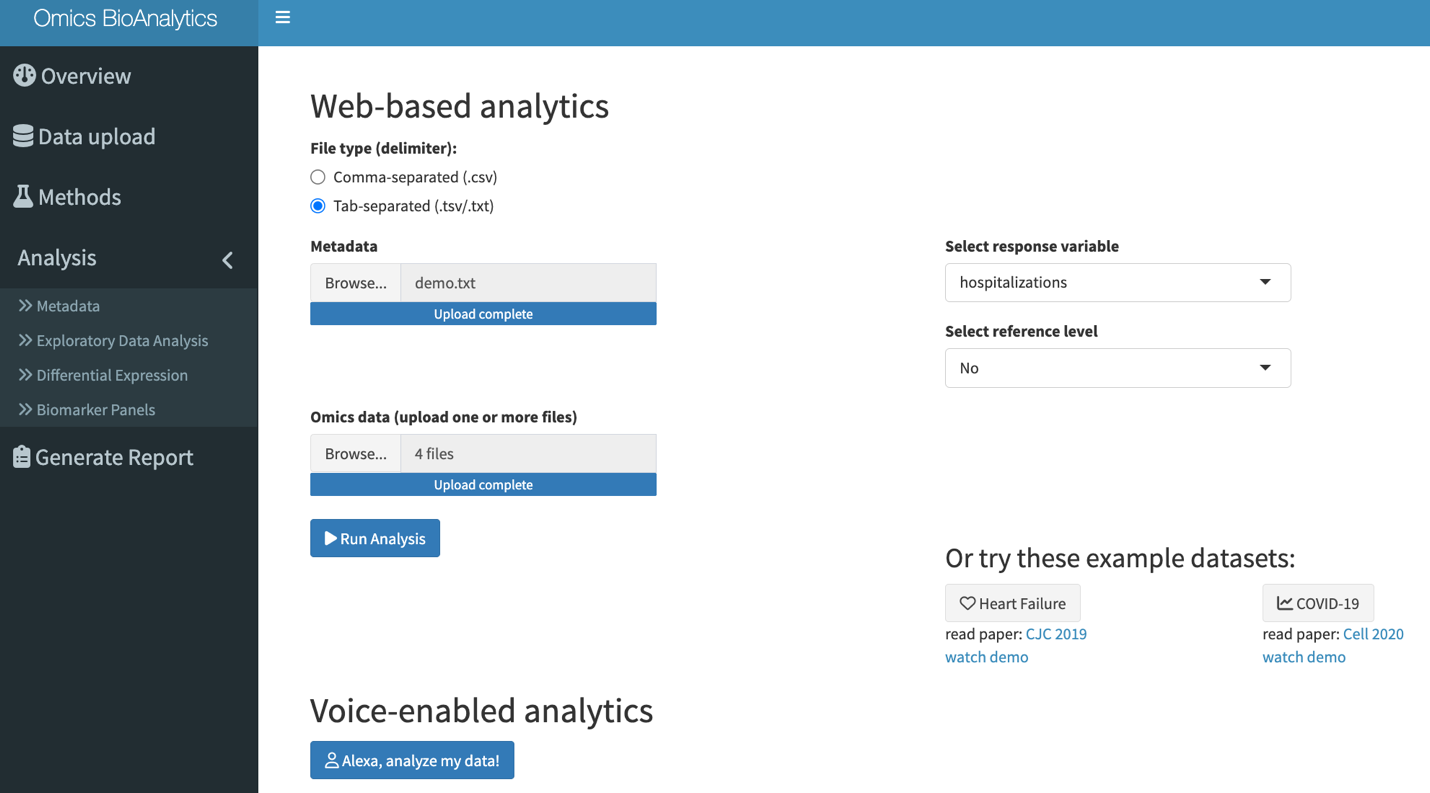


**Supplementary Figure S6. Data upload of heart failure datasets.** Metadata of the samples can be uploaded which prompts the user to select a response variable as well as a reference category. In this case, a demographics dataset was uploaded, and hospitalization status was chosen from the list of categorical variables with two or more categories. One or more omics datasets can also be uploaded, in this case four text files named *cells, holter*, *mrna,* and *proteins* were uploaded. All files have samples along the rows and variables along the columns.

1. **Metadata Analysis**


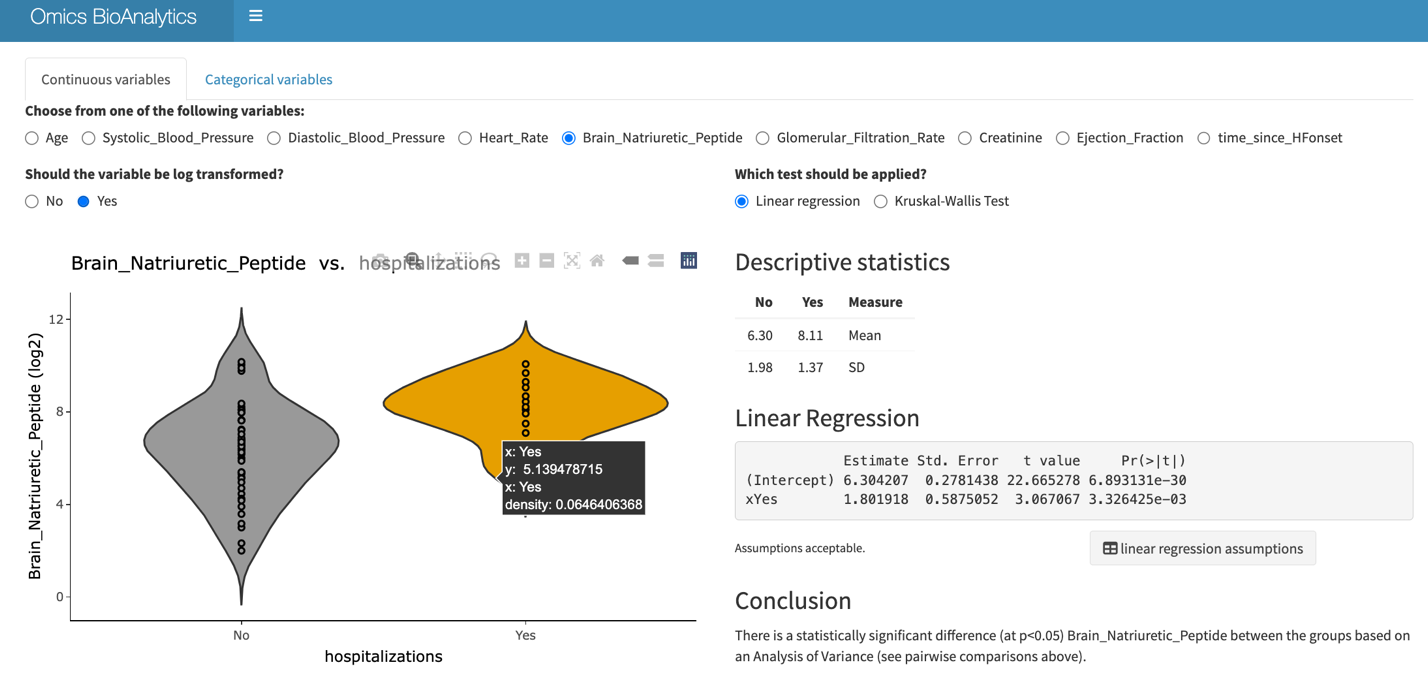


**Supplementary Figure S7. Analysis of continuous metadata variables.** Continuous variables are automatically identified from the metadata file and displayed as radio buttons for individual exploration. The example shows that the average levels of Brain Natriuretic Peptide (BNP) are significantly higher in patients that were hospitalized as compared to those that were not (p<0.05). The scale has been log2-transformed given the data is highly skewed.


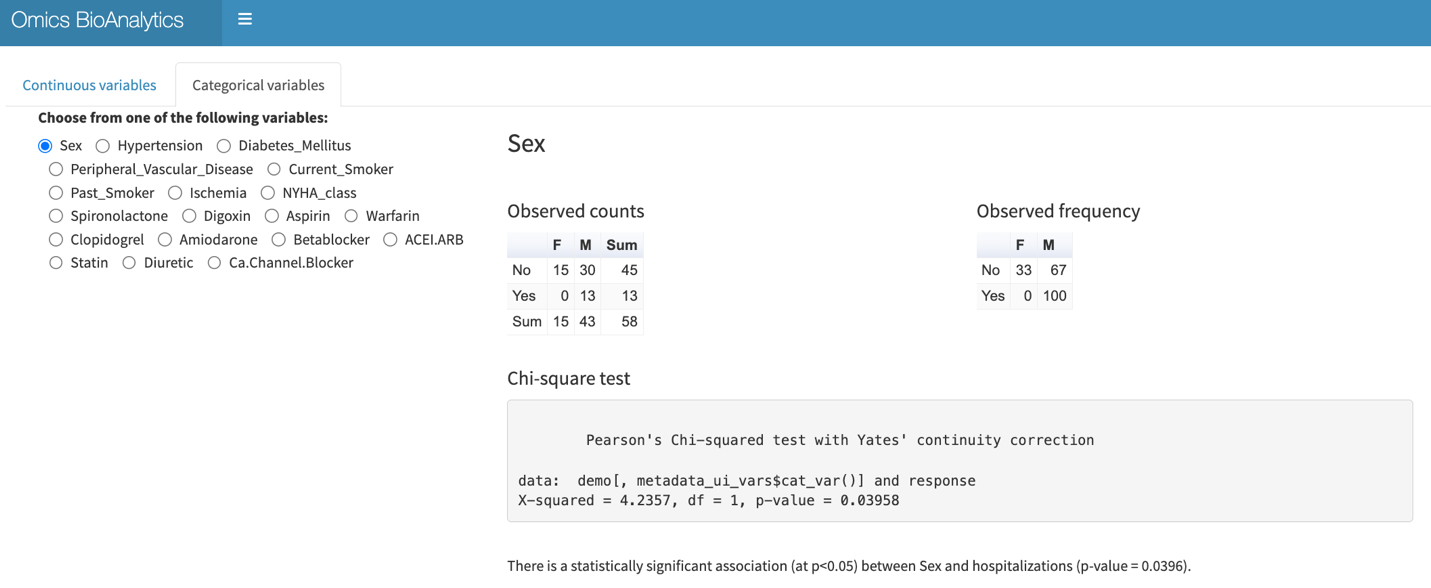


**Supplementary Figure S8. Analysis of categorical metadata variables.** Categorical variables are automatically identified from the metadata file and displayed as radio buttons for individual exploration. The example shows a contingency table between sex and hospitalization status, where a significant association between the observed and expected counts was identified (p = 0.04).

1. **Exploratory Data Analysis**


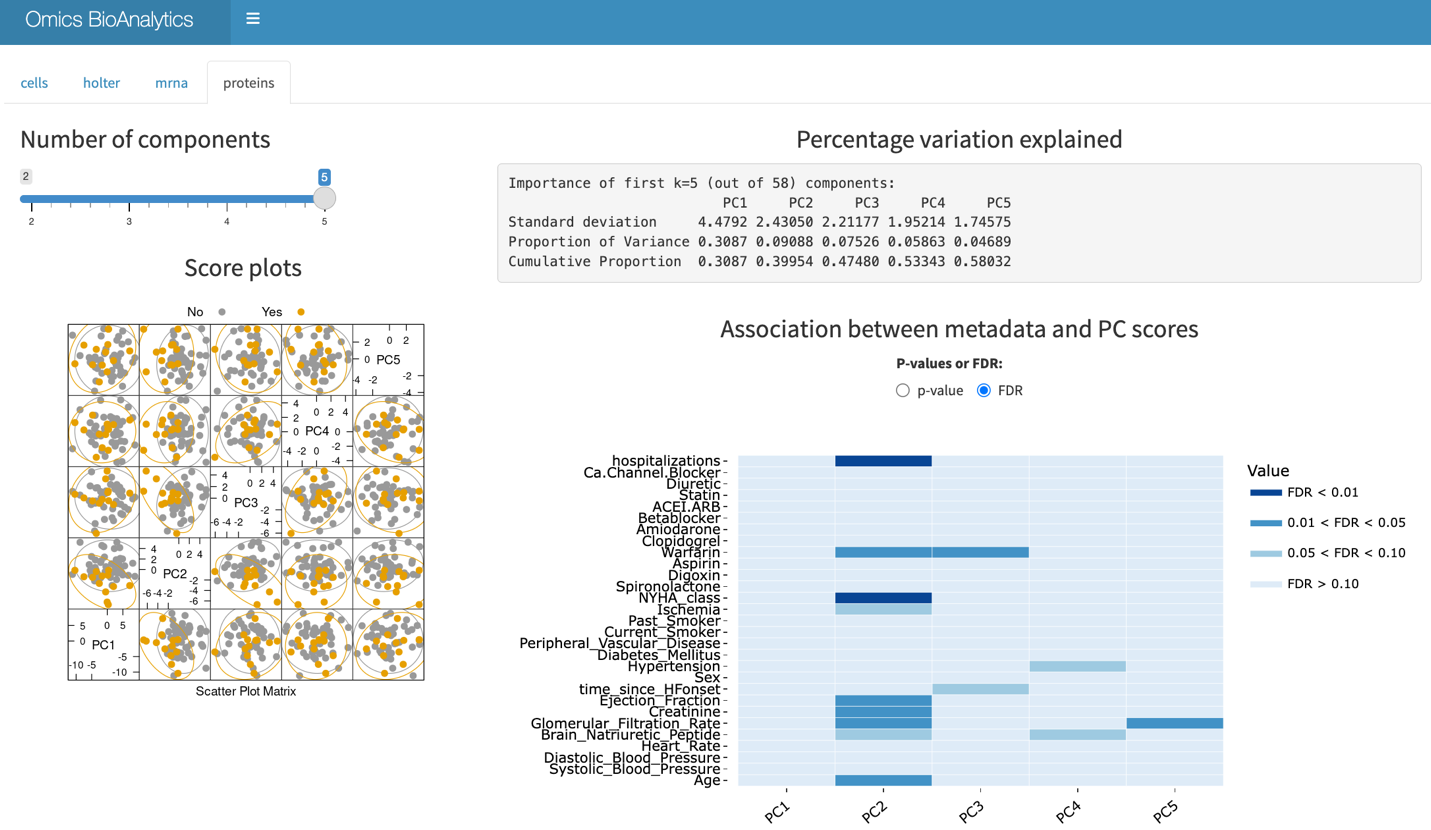


**Supplementary Figure S9. Exploratory data analysis of each uploaded omics dataset.** For each uploaded omics dataset principal component analysis (PCA) is performed. The user can select the number of principal components to depict in the scatter plot matrix. The heatmap depicts the ANOVA corrected p-values, false discovery rate (FDR) comparing each principal component with each metadata variable. The scatter plot matrix (left) depicts the PC scores of the proteomics dataset for the first five principal components, colored by the response variable (hospitalization status) selected during the data upload stage. The heatmap (right) shows the association of each metadata variable with a principal component. For example, hospitalization status is significantly associated with the second principal component (FDR<0.01), which can also be observed in the scatter plot matrix.

1. **Biomarker Discovery Analysis**

**
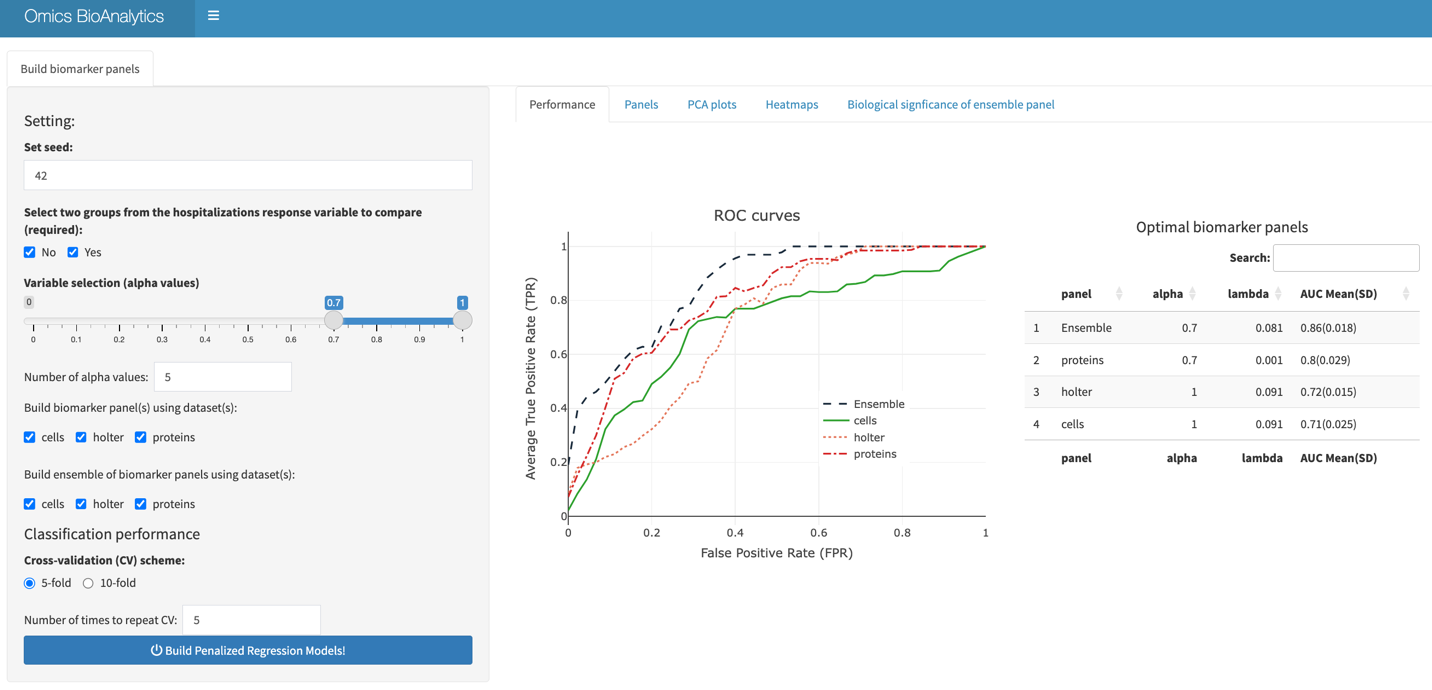
**

**Supplementary Figure S10. Biomarker discovery analysis – Performance tab.** Classification models using penalized regression models (glmnet R-library) are built for each omics dataset for a grid of hyperparameter values [for variable selection (alpha) and shrinkage of regression coefficients (lambda)]. The user can set a grid for the number of variables to select from each omics dataset [alpha=1: LASSO regression (few variables retained in final model), alpha=0: ridge regression (all variables retained in final model)]. The performance is evaluated using 5-fold or 10-fold cross-validation and repeated *k* times (set by user). The user can select the omics datasets for building binary elastic net penalized regression classifiers for and which ones to combine in the ensemble model. The figure shows the datasets that were used to develop individual biomarker panels and the ensemble biomarker panel, the grid used for hyperparameter tuning includes five alpha values from 0.7 to 1 (0.700, 0.775, 0.850, 0.925, 1.000). The average receiver operating characteristic (ROC) curves are depicted with the corresponding summary table, showing that the Ensemble biomarker panel outperforms the individual biomarker panels with an AUC=0.86.


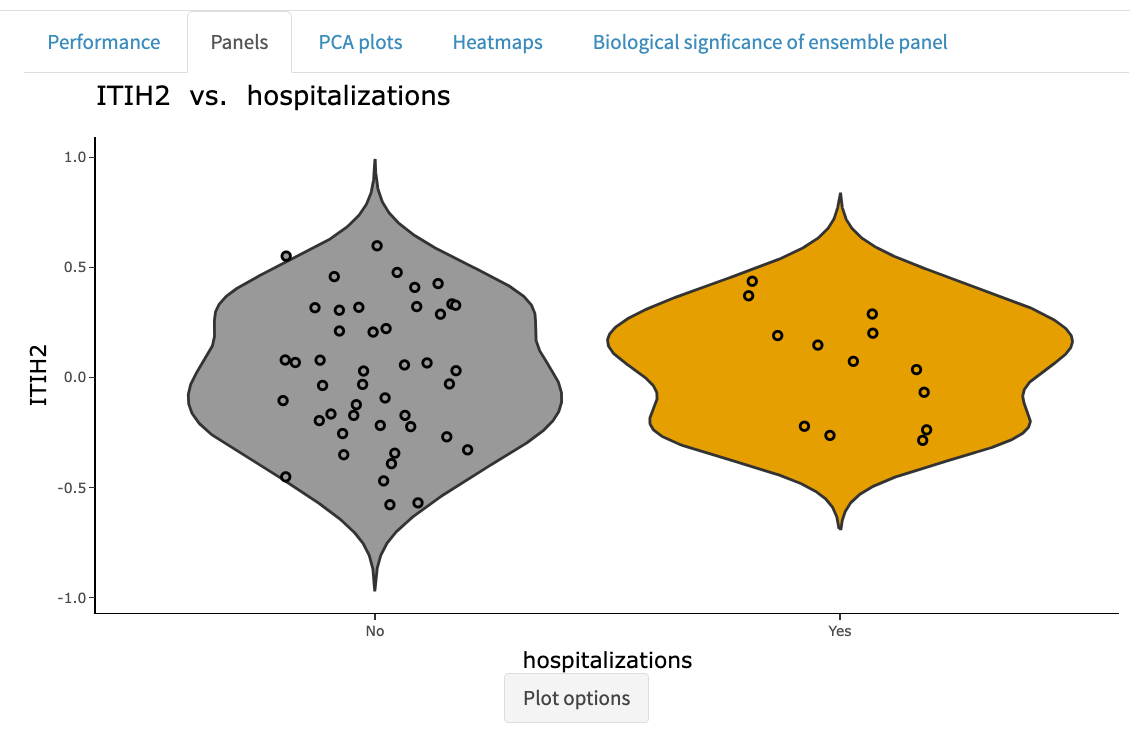


A.


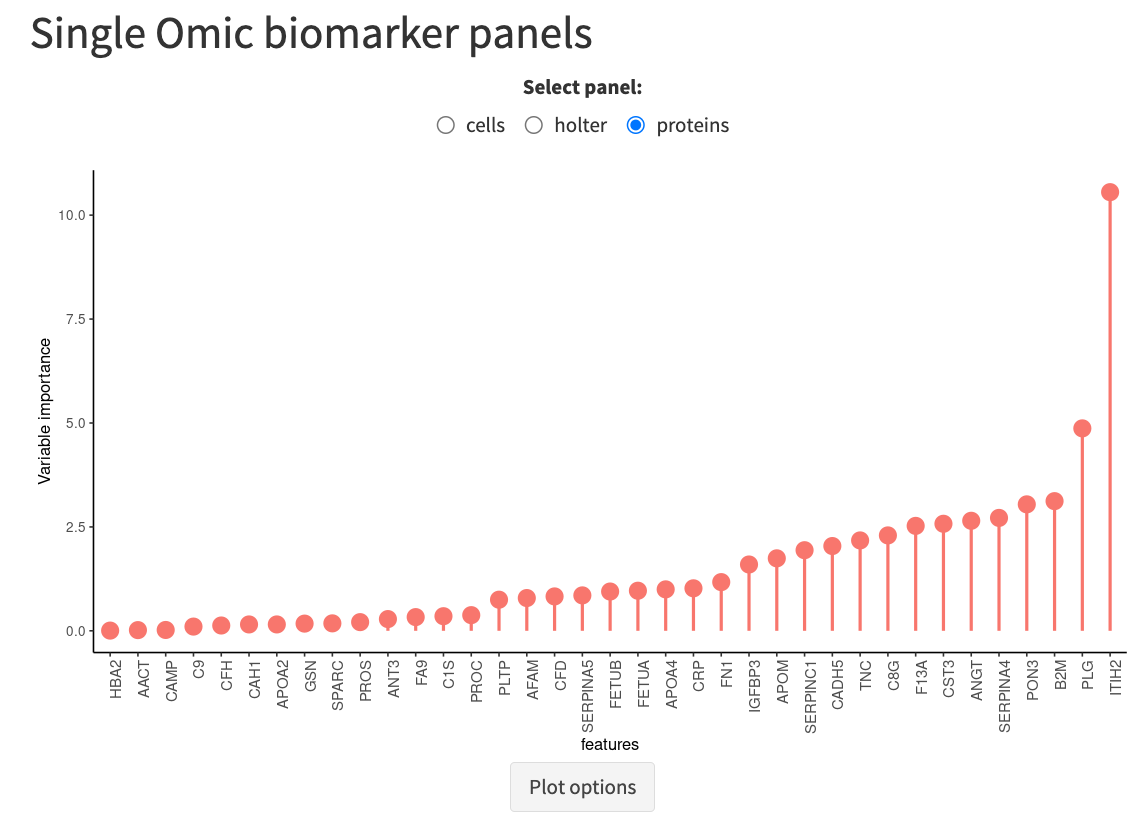


B.

C.


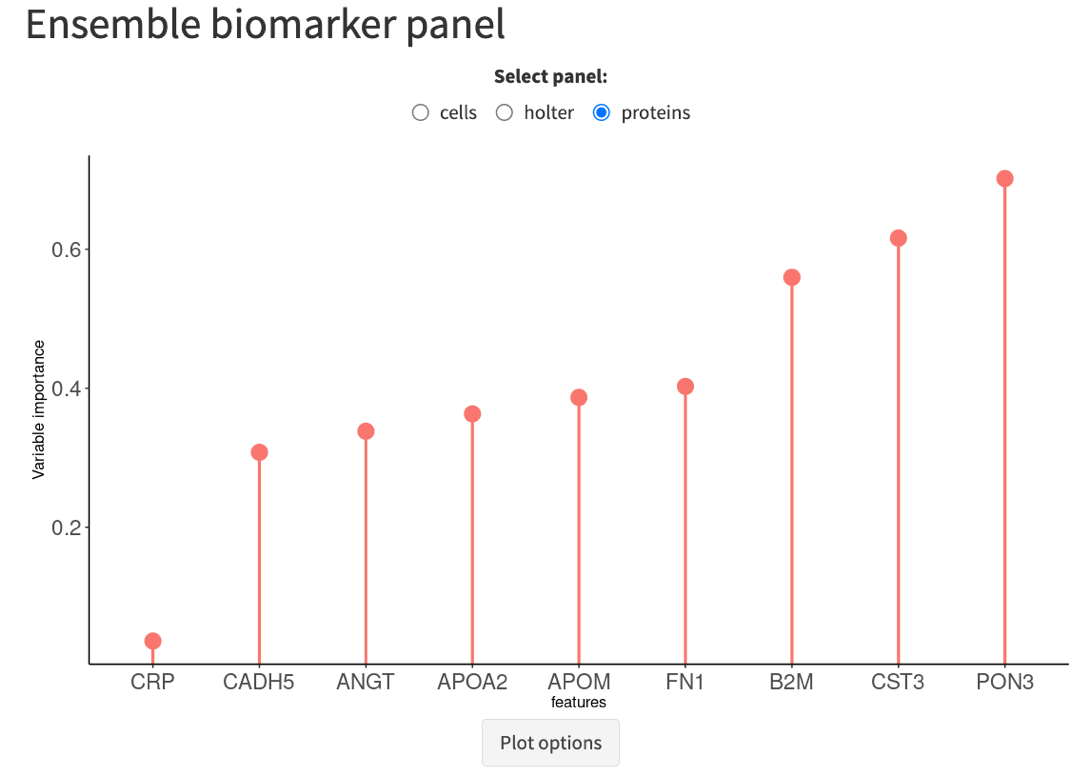


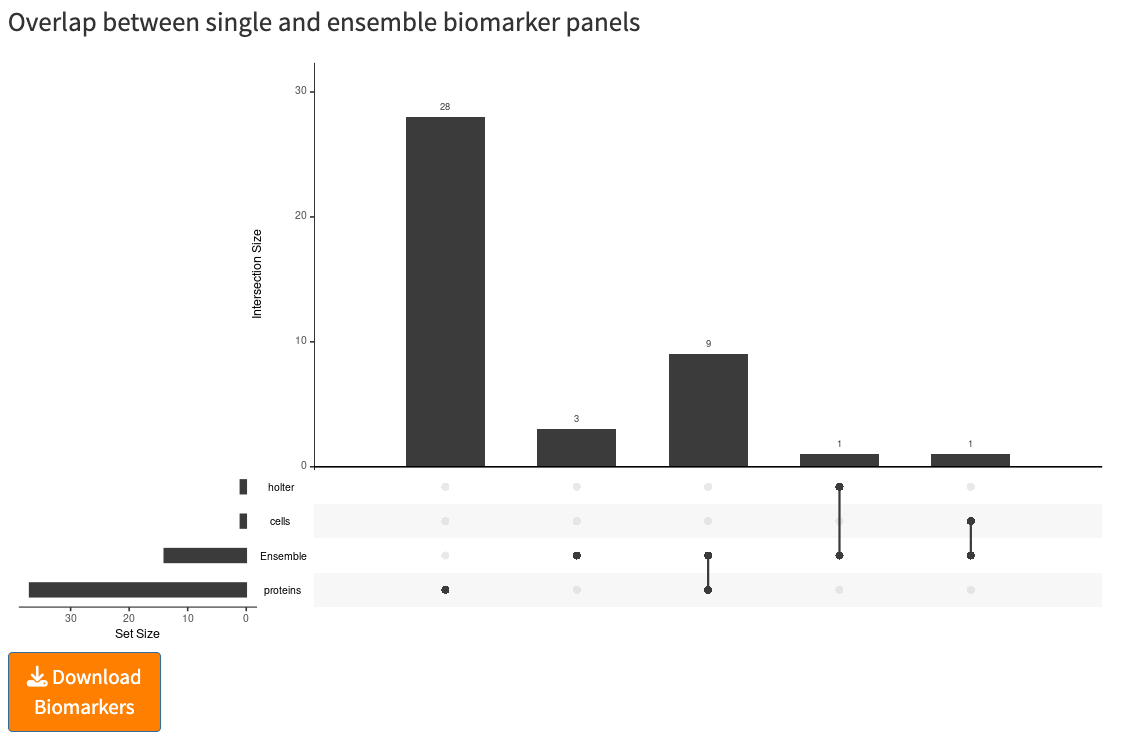


D.

**Supplementary Figure S11.** **Biomarker discovery analysis – Panels tab.** Selected variables in the individual biomarker panels and ensemble biomarker panel are depicted using dot plots. Clicking on a given dot will display the specific variable. In this example, ITIH2 of the protein biomarker panel was clicked and the associated violin plot is displayed. The intersection plot displays the number of overlaps between each pair of panels and, in this example, the greatest overlap is between the ensemble biomarker panel and the mRNA biomarker panel. The list of all biomarkers can also be downloaded.


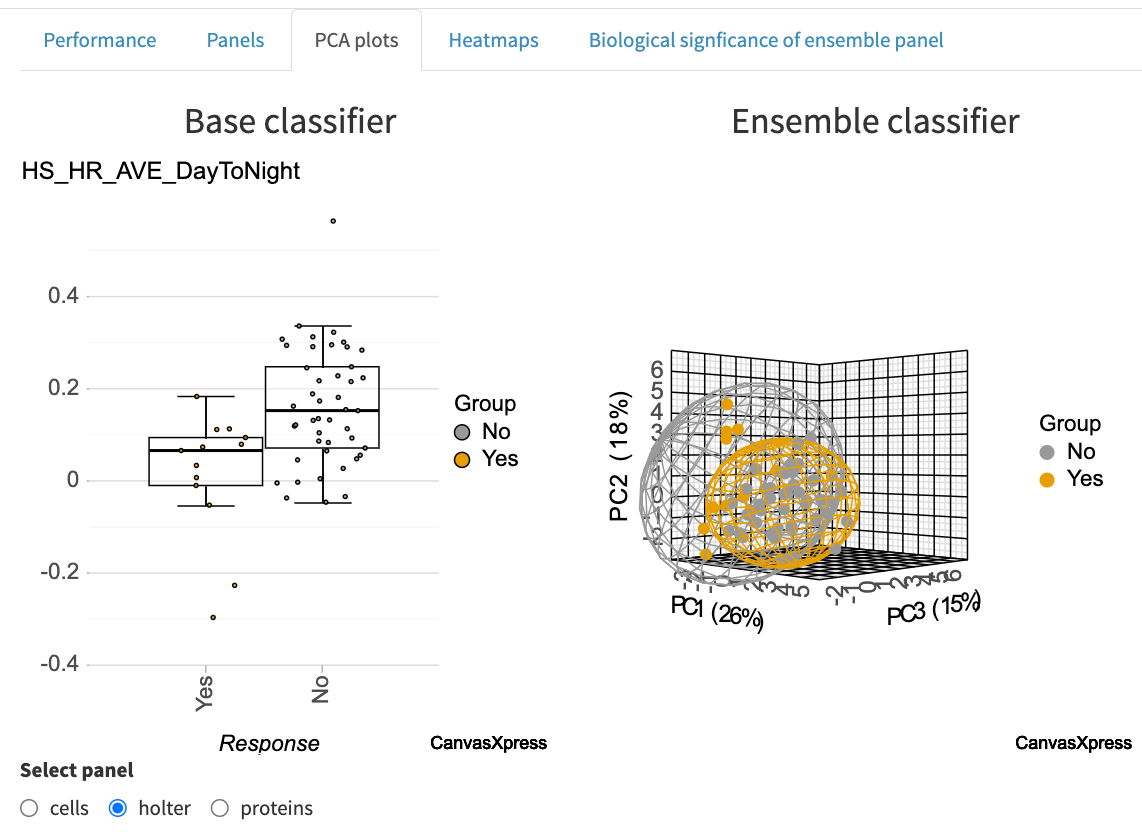


**Supplementary Figure S12.** **Biomarker discovery analysis – PCA plots tab.** The figure displays the PCA score plots using biomarkers in the holter base classifier (left) and using biomarkers of various data types (cells, holter and protein) in the ensemble classifier (right).


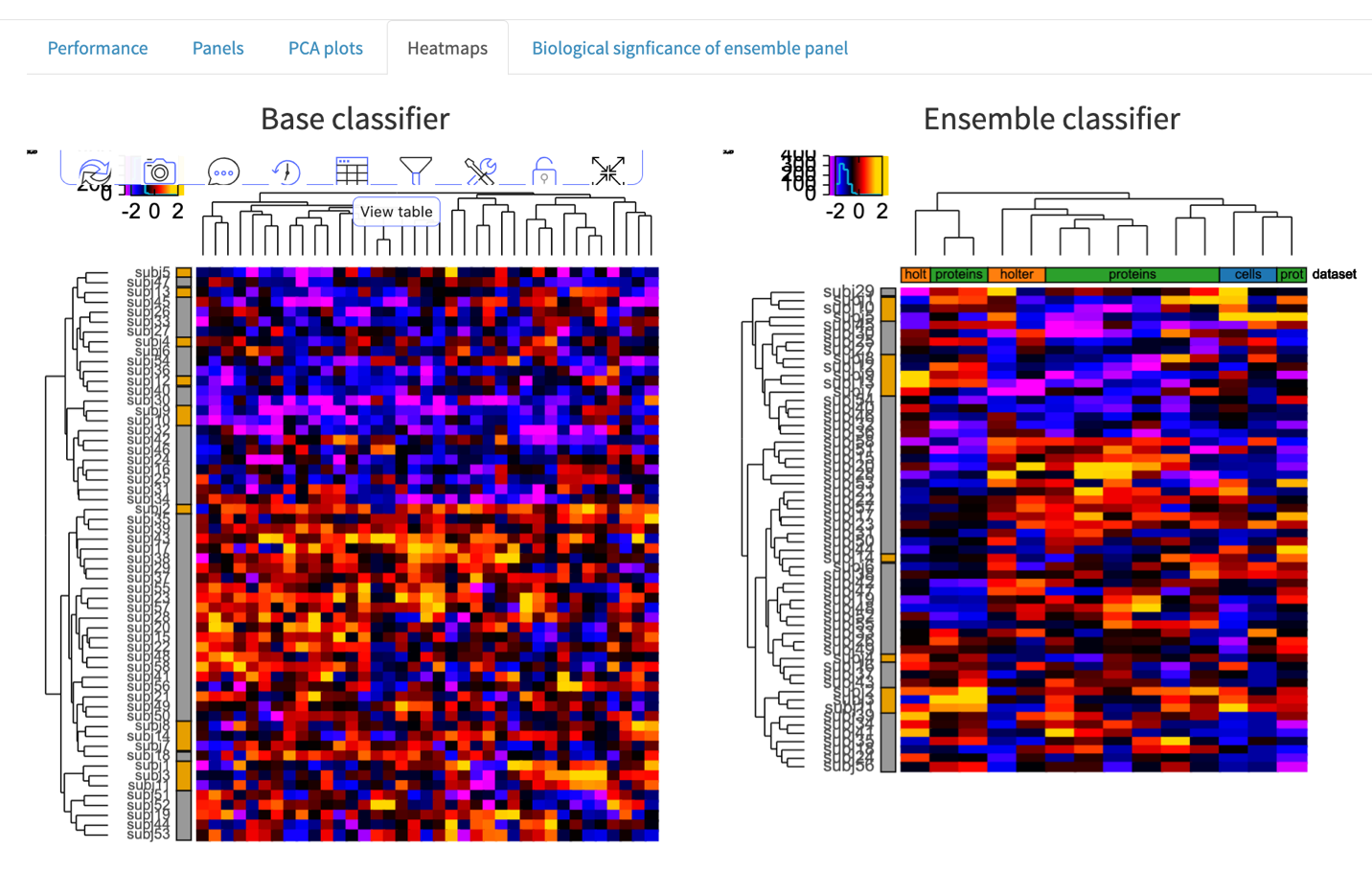


**Supplementary Figure S13.** **Biomarker discovery analysis – Heatmaps tab.** The hierarchical clustering of samples and variables based on the protein biomarkers is displayed using a heatmap on the left whereas biomarkers of various data types (cells, holter, and protein) in the ensemble classifier is displayed on the right. Data is centered, scaled and capped at ±2 prior to plotting. Clicking on the row or column labels of the heatmaps can be used to narrow in on the clustering patterns of specific subgroups or data types. For example, for the ensemble classifier, rows corresponding to samples labelled with a hospitalization status of *Yes* are highlighted.


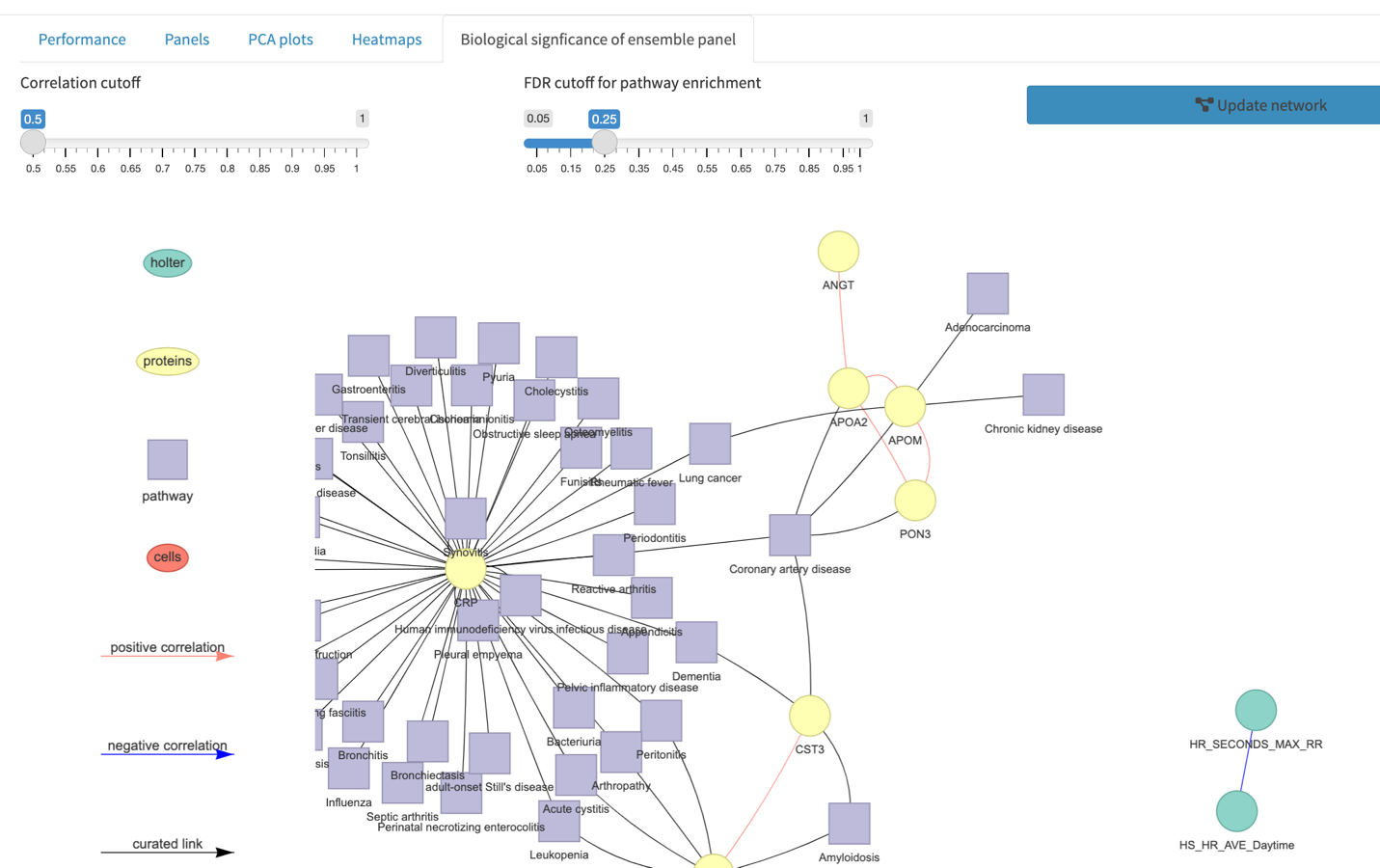


**Supplementary Figure S14.** **Biomarker discovery analysis – Biological significance of ensemble panel tab.** Gene set analysis of variables in the ensemble biomarker panel is performed using various thresholds for significance and pairwise correlations. The sub-network on the right connected proteins (APOM, APOA2, PON3, CRP and CST3) to the *Coronary artery disease* pathway and this network was further extended by including correlated proteins (ANGT and APOA2).

**References**

Blanco-Melo,D. *et al.* (2020) Imbalanced Host Response to SARS-CoV-2 Drives Development of COVID-19. *Cell*, **181**, 1036-1045.e9.

Singh,A., *et al.* (2019) Ensembling Electrical and Proteogenomics Biomarkers for Improved Prediction of Cardiac-Related 3-Month Hospitalizations: A Pilot Study. *Can. J. Cardiol.*, **35**, 471–479.
